# Mass Spectrometry Targeted Assays as a Tool to Improve Our Understanding of Post-translational Modifications in Pathogenic Bacteria

**DOI:** 10.3389/fmicb.2016.01216

**Published:** 2016-08-04

**Authors:** Nelson C. Soares, Jonathan M. Blackburn

**Affiliations:** Division of Chemical and Systems Biology, Department of Integrative Biomedical Sciences, Faculty of Health Sciences, Institute of Infectious Diseases and Molecular Medicine, University of Cape TownCape Town, South Africa

**Keywords:** post-translational modification, protein phosphorylation, proteomics, phosphoproteomics, targeted proteomics, mass spectrometry, PRM, SRM

## Advances in mass spectrometry-based proteomics impact the analysis of bacterial PTMs

Post translational modifications (PTMs) play a vital role in maintaining protein function, regulation, signaling, and many other important cellular processes (Anonsen et al., 2012). For several years, protein PTMs were thought to be unique to eukaryotes; only recently it has been demonstrated that bacteria also contain a wide variety of PTMs including those previous described in eukaryotic cells, for example Ser/Thr/Tyr phosphorylation, lysine-acetylation, glycosylation, ubiquitination, and glutathionylation, as reviewed (Macek and Mijakovic, 2011; Soufi et al., 2012; Michard and Doublet, 2015; Ravikumar et al., 2015). PTMs have been linked to numerous essential bacterial cellular events, including cell division, morphology, and more recently they have also been associated with: Pathogenicity (Michard and Doublet, 2015; Ravikumar et al., 2015); virulence (Lin et al., 2009; Calder et al., 2015; Fortuin et al., 2015); and drug resistance (Soares et al., 2014; Lai et al., 2016). The employment of powerful new proteomics platforms has contributed to significant insight in to the regulatory functions of bacterial PTMs. More than a decade ago, the first attempts to catalog bacterial phosphoproteomes were performed using 2D gel electrophoresis-based (2-DE) approaches, first combined with ^33^P radiolabelling and later with phosphoprotein-specific dyes (Cortay et al., 1986; Bendt et al., 2003; Lévine et al., 2006). However, such studies were limited by a number of technical challenges, including those inherent to gel based approaches (Pietrogrande et al., 2003; Monteoliva and Albar, 2004). Consequently, at the time few phosphoproteins and even fewer phosphorylation sites were reported. As the field of proteomics progressed from 2-DE gel based methodologies to modern liquid chromatography mass-spectrometry (LC-MS/MS) based workflows, significant advances in the analysis of bacterial PTMs were then achieved. The introduction of modern mass spectrometry (MS), specifically the inclusion of Orbitrap mass spectrometers, brought new momentum to the field. In 2006, Mann and colleagues proposed a gel-free approach to explore the phosphoproteome of eukaryotic cells in depth. The study reported the identification of more than 6600 phosphorylation sites (Olsen et al., 2006). Their workflow involved the enzymatic digestion of complex protein extracts into peptides and then phosphopeptides were enriched by a combination of strong cation exchange and TiO_2_ chromatography. Macek et al. (2007) used similar technology to describe a site–specific, *in vivo* phosphoproteome of a gram positive model organism, *Bacillus subtilis*. The study represented a main breakthrough in the bacterial phosphoproteomic field: the number of reported *B. subtilis* phosphoproteins went from 16 phosphorylation sites on eight proteins to 103 unique phosphopetides on 78 proteins (Macek et al., 2007). This order of magnitude jump in the field then inspired many other subsequent studies that successfully applied variants of the protocol described by Macek et al. (2007), in particular those that employed phosphopetide enrichment via immobilized metal affinity chromatography (IMAC; Thingholm and Jensen, 2009) to perform high throughput analysis of different bacterial phosphoproteomes (Ravichandran et al., 2009; Qu et al., 2013). Today, several bacterial phosphoproteomes have been successfully characterized including some important human pathogenic bacteria: *Pseudomonas aeruginosa* (Ravichandran et al., 2009); *Klebsiella pneumoniae* (Lin et al., 2009); mycobacterium spp. (Prisic et al., 2010; Fortuin et al., 2015; Nakedi et al., 2015; Calder et al., 2016); *Helicobacter pylori* (Ge et al., 2011), streptomyces spp. (Manteca et al., 2011); *Acinectobacter baummnii* (Soares et al., 2014; Lai et al., 2016); *Staphylococcus aureus* (Basell et al., 2014); and Enteropathogenic *Escherichia coli* (Scholz et al., 2015). The development of new high throughput mass spectrometric instrumentation (Michalski et al., 2011b, 2012; Scheltema et al., 2014; Erickson et al., 2015) together with continuous optimization of LC-MS/MS shotgun proteomics workflows (Michalski et al., 2011a; Kelstrup et al., 2012; Pirmoradian et al., 2013) resulted in increased resolution, sensitivity, and fragmentation and has contributed to the successful identification and localisation of phosphosites. Such approaches have also now allowed for the identification of other bacterial PTMs such as lysine acetylation (Zhang et al., 2009; Weinert et al., 2013; Wu et al., 2013; Liu et al., 2014; Ouidir et al., 2015; Xie et al., 2015), ubiquitination (Valkevich et al., 2014), and glycosylation (Scott et al., 2011; Anonsen et al., 2012; Smith et al., 2014). It is therefore well established that large scale LC-MS/MS shotgun proteomics is the technique of choice for identifying bacterial post-translationally modified peptides/proteins.

When quantifying the state of a modified peptide/protein under a certain experimental condition(s) and/or during different cellular processes, it is important to determine its regulatory function and ultimately link this to a specific molecular mechanism. Recent advances in quantitative LC-MS/MS based proteomics have made possible the reliable quantification of thousands of phosphorylation peptides in eukaryotic cells (Pan et al., 2008) and, likewise, a number of studies have successfully applied different quantification methods to describe the dynamics of bacterial phosphoproteomes under different cellular states or experimental conditions. For instance, stable isotope labeling by amino acids in cell culture (SILAC) has recently been applied to establish the dynamics of both the proteome and phosphoproteome of *E. coli* during five different growth phases in liquid cell culture (Soares et al., 2013). In that study, 76 Ser/Thr/Tyr phosphorylation events were quantified in all growth phases. Importantly the use of SILAC enabled the measurement of median occupancies of phosphorylation sites, which, unlike eukaryotic cells (Pan et al., 2008), were generally low (< 12%) (Soares et al., 2013). SILAC has been successfully used on a number of occasions to determine the varying levels of Ser/Thr/Tyr phosphorylation events. However, the issues associated with SILAC when applied to bacteria are well documented (Soufi and Macek, 2014) and include, in particular, cases such as mycobacteria where there is no available viable, lysine-deficient mutant. Alternatively, chemical labeling, such as peptide demethylation labeling (Boersema et al., 2009), has been proven a convenient and efficient method to obtain differential quantification of bacterial PTMs (Spat et al., 2015; De Keijzer et al., 2016) and has gained increasing popularity. Additionally, an always attractive quantitative approach is label free quantification (LFQ), which has been applied on a number of occasions to further investigate the different PTMs levels in bacteria. Within this context, although phospho-enrichment strategies combined with LFQ analyses look promising, it has been pointed out (Rosenberg et al., 2015) that low occupancy among bacterial phosphorylation events may compound possible inconsistences amongst biological replicates.

MS based detection of PTMs is normally based on a mass shift introduced by the site-specific modification, however these are often sub-stoichiometric and usually occur at low abundance, thus an enrichment process (such as those mentioned above) is typically carried out for a specific PTM prior to subsequent MS analysis. The enrichment steps are however, for many, a major drawback in bacterial PTM analyses, since the enrichment step typically requires large amounts of starting material including protein concentrations in the order of 5–9 mg (Soufi et al., 2008; Soares et al., 2013, 2014; Spat et al., 2015). While this is can be easily achieved when using liquid cell culture, this may represent a major technical challenge for other studies, such those looking at host pathogen interactions at the site of infection, where it is virtually impossible to isolate the large number of bacterial cells or the physical amount of protein needed in those protocols. Additionally, the multiple rounds of enrichment steps remain a source of possible technical variations that can bias downstream quantitative analyses. Ultimately, the efficiency and reproducibility of the enrichment protocol across multiple samples is determinant both in terms of coverage as well as in terms of accurate quantification.

## MS based targeted methodologies will allow detection and quantification of low abundant bacterial PTMs

Emerging targeted MS methodologies, namely selected reaction monitoring (SRM), and parallel reaction monitoring (PRM), appear as perhaps the latest breakthrough within the field. Specifically, targeted mass spectrometry offers unequaled capability to characterize and quantify a specific set of proteins/peptides reproducibility, in any biological sample. In this hypothesis-driven approach (Picotti et al., 2013), only a small number of peptides are used as surrogate markers for the protein of interest, which are selectively measured in predefined m/z ranges and retention time windows. SRM (often called MRM, multiple reaction monitoring) is a targeted approach to quantitate pre-selected peptides by monitoring specific precursor-to-product ion transitions in a triple-quadrupole mass spectrometer (Picotti et al., 2010, 2013). While SRM measurements use a low resolution MS instrument, PRMs alternatively benefit from the capabilities of high resolution quadrupole and Orbitrap analysers (Peterson et al., 2012). The increased quality of hybrid quadrupole-Orbitrap analyses has consequently improved the quality of the target methods, mainly in terms of accuracy at the level of fragment, which enables the fundamental distinction of target compounds from the undesired background ions and enables a significantly lowered detection limit. In mammalian systems, SRM assays have been successfully applied in a large scale targeted proteomics experiment of a phosphorylation network. In the first of its kind study, Wolf-Yadlin et al. (2007) applied SRM to monitor the dynamics over time of tyrosine phosphorylation events in EGFR signaling after EGF stimulation. The authors used a combination of iTRAQ peptide labeling and phosphotyrosine peptide immune-purification coupled with IMAC to enrich for phosphotyrosine containing peptides (Wolf-Yadlin et al., 2007). More recently, Adachi et al. (2016) described the application of a large-scale phosphoproteome analysis and SRM-based quantification to develop a strategy for the systematic discovery and validation of biomarkers. Their two step approach described uses a typical IMAC based phosphoenrichment coupled to shotgun MS-based proteomic analysis in order to identify differentially modulate phosphopeptides; identified candidates are then validated by SRM analysis (Adachi et al., 2016). In another recent, well-designed application, Altelaar and co-workers suggested a strategy to monitor signal transduction pathways: By combining targeted quantitative proteomics with high selective phosphopeptide enrichment, the authors monitored the phosphorylation dynamics of the PI3K-mTOR and MAPK signaling network (De Graaf et al., 2015). Interestingly, in order to increase the success rate of the phosphopeptide SRM assays the authors focused on phosphopeptides reported previously by the group and from publicly available shotgun proteomics experimental data. The rationale for this approach is that phosphorylation alters the local charge distribution, which may interfere with proteolytic cleavage, thus previous knowledge of the phosphosite would enable a more efficient SRM assay. The study used synthetic stable isotope standard phosphopeptides for increased accuracy in quantification (De Graaf et al., 2015). Despite all this, measuring PTMs events through SRM can be challenging and in most cases involves time consuming assay optimization and reliance on synthetic peptide standards (Lawrence et al., 2016), which can be somewhat discouraging in practice. However, recently Lawrence et al. have proposed the use of PRMs as a straightforward and efficient means for large-scale targeted phosphoproteomic analysis. Here, instead of using databases of previously reported human phosphopeptide sequences, the authors report a highly comprehensive shotgun global phosphoproteomic analysis that resulted in the identification of more than 7.5 million phosphopetide spectral matches corresponding to 109,611 phosphorylation sites (Lawrence et al., 2016). Subsequently, the authors used the generated information, including the respective phosphopeptides retention times, to develop a plug-and-play phosphoproteomic system, based on sets of targeted, label-free phosphopeptide PRM assays in order to interrogate the dynamics of the IGF1/AKT signaling pathway. Importantly, the study demonstrated that without sample fractionation and or phosphopeptide enrichment (Lawrence et al., 2016), PRM label-free quantification assays is a rapid assay for measuring virtually any known phosphorylation event in the human species.

Targeted methodologies have been successfully applied to investigate aspects of the pathogenic bacteria proteome (Lange et al., 2008; Karlsson et al., 2012; Schubert and Aebersold, 2015; Schubert et al., 2015; Peters et al., 2016). A noteworthy highlight is that Ruedi Aebersold and colleagues have recently generated a library of targeted SRM assays for ~97% of the 4012 annotated *Mycobacterium tuberculosis* proteins and were able to reproducibly quantify ~72% of the theoretical *M. tuberculosis* proteome in single unfractionated runs on a triple quadrupole MS (Schubert et al., 2013). As such, the generated *M. tuberculosis* proteome library represents a valuable experimental resource that now in theory enables researchers to interrogate and quantify essentially the entire proteome of *M. tuberculosis* in a single experiment. This allows for greater understanding of, for example, differences in expressed pathogenicity and virulence between clinical isolates. However, in order to further investigate mycobacterial or any other bacterial PTMs in a large scale, targeted and quantitative manner, it will be necessary first to follow a workflow similar to that used in mammalian cells as suggested by Lawrence et al. (2016). A strategy based on previous knowledge of the post translationally modified proteins/peptide is sensitive enough to detect low abundance, modified peptides. To date, much of the work on pathogenic bacteria using MS-based proteomics has focused on cataloging, and in some cases quantifying, PTMs that occur under *in vitro* conditions. Although informative, such reports come with the caveat of the studied conditions often being detached from the unique microenvironment that pathogenic bacteria are exposed to during host infection and/ or during colonization (e.g., inside the macrophage). Thus, in order to gain meaningful insights into the dynamics and role of different bacterial PTMs during host-pathogen interactions, it is crucial that researchers in the field join efforts to once again take advantage of recent advances in mass spectrometry instrumentation and develop targeted, quantitative PTM workflows that enable detection and accurate quantification of low-abundance, modified analytes, ideally circumventing the need for additional PTM enrichment steps in the process.

## Author contributions

NS wrote the original draft of this paper read, and edit final draft. JB critically discussed the content of the manuscript and provided editing support.

## Funding

This work was supported by a research grant from the National Research Foundation of South Africa (NRF; grant number 98963 and 95984). The authors have no other relevant affiliations or financial involvement with any organization or entity with a financial interest in or financial conflicts with subject matter or materials discussed in the manuscript.

### Conflict of interest statement

The authors declare that the research was conducted in the absence of any commercial or financial relationships that could be construed as a potential conflict of interest.
